# High glucose suppresses autophagy through the AMPK pathway while it induces autophagy via oxidative stress in chondrocytes

**DOI:** 10.1038/s41419-021-03791-9

**Published:** 2021-05-18

**Authors:** Ben Wang, Yifeng Shi, Jiaoxiang Chen, Zhenxuan Shao, Libin Ni, Yan Lin, Yaosen Wu, Naifeng Tian, Yifei Zhou, Liaojun Sun, Aimin Wu, Zhenghua Hong, Xiangyang Wang, Xiaolei Zhang

**Affiliations:** 1 Department of Orthopaedics, The Second Affiliated Hospital and Yuying Children’s Hospital of Wenzhou Medical University, Wenzhou, 325027 Zhejiang China; 2 Key Laboratory of Orthopaedics of Zhejiang Province, Wenzhou, 325027 Zhejiang China; 3 The Second School of Medicine, Wenzhou Medical University, Wenzhou, 325027 Zhejiang China; 4 Department of Orthopaedics, Taizhou Hospital Affiliated of Wenzhou Medical University, Linhai, 317000 China; 5 Chinese Orthopaedic Regenerative Medicine Society, Hangzhou, Zhejiang China

**Keywords:** Autophagy, Bone, Endocrine system and metabolic diseases

## Abstract

Diabetes (DB) is a risk factor for osteoarthritis progression. High glucose (HG) is one of the key pathological features of DB and has been demonstrated to induce apoptosis and senescence in chondrocytes. Autophagy is an endogenous mechanism that can protect cells against apoptosis and senescence. The effects of HG on autophagy in cells including chondrocytes have been studied; however, the results have been inconsistent. The current study aimed to elucidate the underlying mechanisms, which could be associated with the contrasting outcomes. The present study revealed that HG can induce apoptosis and senescence in chondrocytes, in addition to regulating autophagy dynamically. The present study demonstrated that HG can cause oxidative stress in chondrocytes and suppress the AMPK pathway in a dose-dependent manner. Elimination of oxidative stress by Acetylcysteine, also called *N*-acetyl cysteine (NAC), downregulated autophagy and alleviated HG-stimulated apoptosis and senescence, while activation of the AMPK signaling pathway by AICAR not only upregulated autophagy but also alleviated HG-stimulated apoptosis and senescence. A combined treatment of NAC and AICAR was superior to treatment with either NAC or AICAR. The study has demonstrated that HG can suppress autophagy through the AMPK pathway and induce autophagy via oxidative stress in chondrocytes.

## Introduction

Osteoarthritis (OA) is a prevalent and irreversible disease of the joints, which causes considerable individual suffering and a heavy social burden globally^1^. The primary features of OA are articular cartilage disruption, subchondral bone remolding, and synovitis, leading to pain, stiffness, and motor disability among others. The occurrence and development of OA is influenced by various risk factors such as diabetes (DB), heredity, obesity, and trauma^2–4^. Numerous studies have established that DB is a key factor that adversely influences the onset of OA and exacerbates OA symptoms^5–7^. According to the American Diabetes Association, DB is a type of metabolic disease characterized by hyperglycemia due to defects in insulin secretion, insulin action, or both^4^. Chronic hyperglycemia of DB may trigger long-term joint damage and dysfunction. Previous studies on articular tissues have demonstrated the adverse influence of DB on biochemical and biomechanical changes in joints and described the pathological manifestation and molecular mechanisms activated in high-glucose environments, which could cause OA^8–13^. With the increasing prevalence of DB and OA^14,15^, an increasing number of researchers have begun to focus on the precise molecular mechanisms of DB on OA, although the pathogenesis and underlying mechanisms have not been completely understood.

Autophagy is an intracellular degradation mechanism that plays a critical role in maintaining cellular integrity and survival by eliminating unnecessary proteins and impaired organelles^16,17^. Autophagy is a key process in normal articular cartilage; therefore, its disease-related loss is closely associated with OA^18,19^. Autophagy dysfunction can be attributed to the effect of high glucose (HG), with a low rate of cell turnover and poor regenerative capacity^19,20^. A few studies on the association between autophagy and DB-related diseases have been performed. For example, a previous study conducted by our research group revealed that DB can cause intervertebral disc degeneration by accelerating apoptosis and senescence of nucleus pulposus cells and that autophagy as a response mechanism can be upregulated in diabetic rats with degenerated intervertebral discs^21^. In OA, a converse outcome of defective autophagy was observed in diabetic patients with OA and experimental mice, suggesting that HG could compromise autophagy capacity in diabetic patients with OA^22,23^. The studies revealed contrasting results for in vivo experiments because the effects of DB on autophagy rather than HG could be attributed to several factors such as aging, inflammation, vascular injury, etc^24,25^. Notably, in vitro studies attempted to determine whether HG led to inhibition or activation of autophagy. HG resulted in decreased LC3II levels and elevated p62 levels in vascular smooth muscle cells^24^; similarly, inhibition of HG-mediated autophagy was observed in the human neuroblastoma cell lines^25^. By contrast, Zhao et al. established that excessive autophagy in H9c2 cells was induced in a HG medium^26^. Furthermore, two studies reported that high concentrations of glucose facilitated autophagy activation in trophoblast cell lines and the cardiomyocyte cell lines^27,28^. To the best of our knowledge, the effects of HG on autophagy in chondrocytes remain indeterminate.

Therefore, the aim of the present study was to investigate the effects of HG on autophagy and related mechanisms using various concentrations of HG to stimulate primary articular chondrocytes and to provide a novel and promising strategy for preventing DB-induced OA.

## Results

### Effects of HG on autophagy, apoptosis, and senescence in chondrocytes

**Fig. 1:**
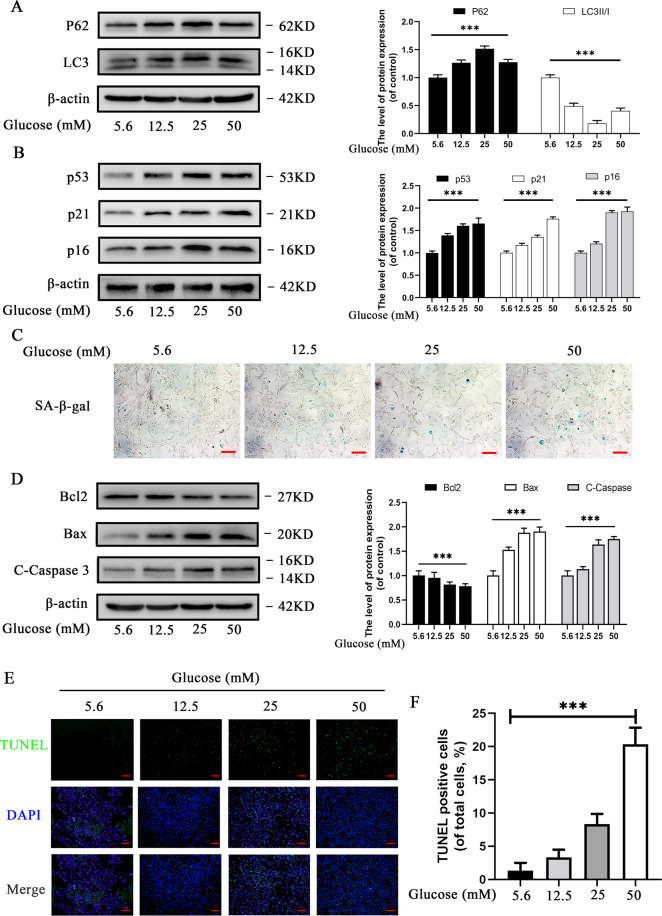
Effects of high glucose (5.6, 12.5, 25, and 50 mM) on autophagy, apoptosis, and senescence in chondrocytes. **A** Western blot results and quantification analyses of p62, and LC3 in chondrocytes under various glucose concentrations. **B** Western blot results and quantification analyses of p53, p21, and p16 treated with various glucose concentrations. **C** Representative micrographs of SA-β-gal staining representing the effects of glucose on senescence. Scale bar 100 μM. **D** Western blot results and quantification analyses of Bcl2, Bax, and cleaved caspase-3 treated with various glucose concentrations. **E** Representative micrographs of TUNEL staining representing the effects of glucose on apoptosis. **F** TUNEL-positive cell intensity under various glucose concentrations. Scale bar = 200 μM. Data are presented as means ± SD (*n* = 5). ^*^P < 0.05, ^**^P < 0.01^,^ and ^***^P < 0.001.

Cells were treated with various concentrations of glucose (5.6, 12.5, 25, and 50 mM) for 24 h to investigate the effects of HG on chondrocytes. The effect of HG on autophagy was assessed by Western blot and the results revealed that p62 level was remarkably upregulated, whereas LC3II/I level was remarkably downregulated with an increase in the concentration of glucose, suggesting that HG inhibited autophagy (Fig. 1A). However, p62 and LC3II/I levels decreased in chondrocytes treated with 50 mM glucose when compared with chondrocytes treated with 25 mM glucose, suggesting that the effect of HG on autophagy in rat chondrocytes was variable and dose-dependent.

The expression of senescence-related markers (p53, p21, and p16) increased markedly with increase in glucose concentration (Fig. 1B). SA-β-gal staining results revealed that HG significantly increased the number of SA-β-gal-positive cells in a concentration-dependent manner (Fig. 1C). In addition, Western blot analysis revealed that cleaved caspase-3 and Bax (the pro-apoptotic index) increased considerably, while Bcl-2 (the anti-apoptotic index) decreased with increasing glucose concentrations (Fig. 1D). Furthermore, TUNEL assay demonstrated the HG induced apoptosis of chondrocytes in a concentration-dependent manner (Fig. 1E, F). The results suggested that HG not only accelerates senescence but also promotes apoptosis in rat chondrocytes in a concentration-dependent manner.

### HG induces oxidative stress and suppresses AMPK signaling in chondrocytes

HG has been reported to induce cellular damage through oxidative stress^29,30^; HG is a primary source of cellular energy, which may suppress a key energy-sensing pathway, the AMPK pathway^31,32^. Oxidative stress and the AMPK pathway have been demonstrated to be associated with autophagy^33,34^. Therefore, we evaluated the effects of HG on oxidative stress and the AMPK pathway.

**Fig. 2:**
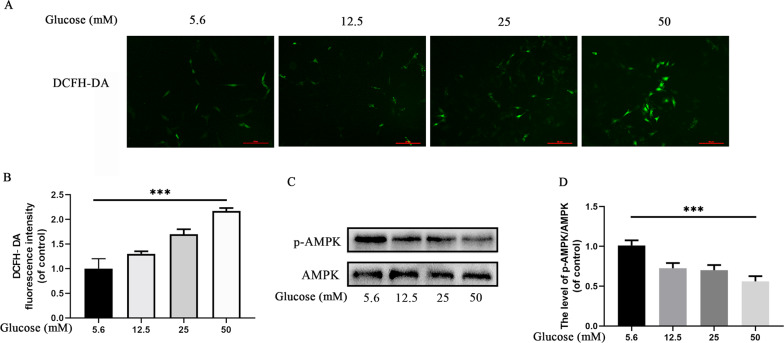
High glucose induces (5.6, 12.5, 25, and 50 mM) oxidative stress and suppresses AMPK signaling in chondrocytes. **A** Representative micrographs of DCFH-DA staining representing the levels of ROS in chondrocytes. Scale bar = 50 μM. **B** DCFH-DA fluorescence intensity of chondrocytes treated with various glucose concentrations. **C** Expression and quantification analyses of p-AMPK and AMPK in chondrocytes treated with various glucose concentrations. **D** Expression of p-AMPK/AMPK under various glucose concentrations. Data are presented as means ± SD (*n* = 5). ^*^P < 0.05, ^**^P < 0.01^,^ and ^***^P < 0.001.

DCFH-DA is a specific probe for intracellular ROS. The DCFH-DA staining assay (Fig. 2A, B) revealed that HG induced higher-ROS levels in a concentration-dependent manner. Western blot results revealed that the ratio of p-AMPK/AMPK decreased in a concentration-dependent manner (Fig. 2C, D). The results suggested that HG can influence chondrocytes by inducing oxidative stress and suppressing AMPK signaling.

### Elimination of oxidative stress suppresses autophagy and inhibits apoptosis and senescence in HG-treated chondrocytes

**Fig. 3:**
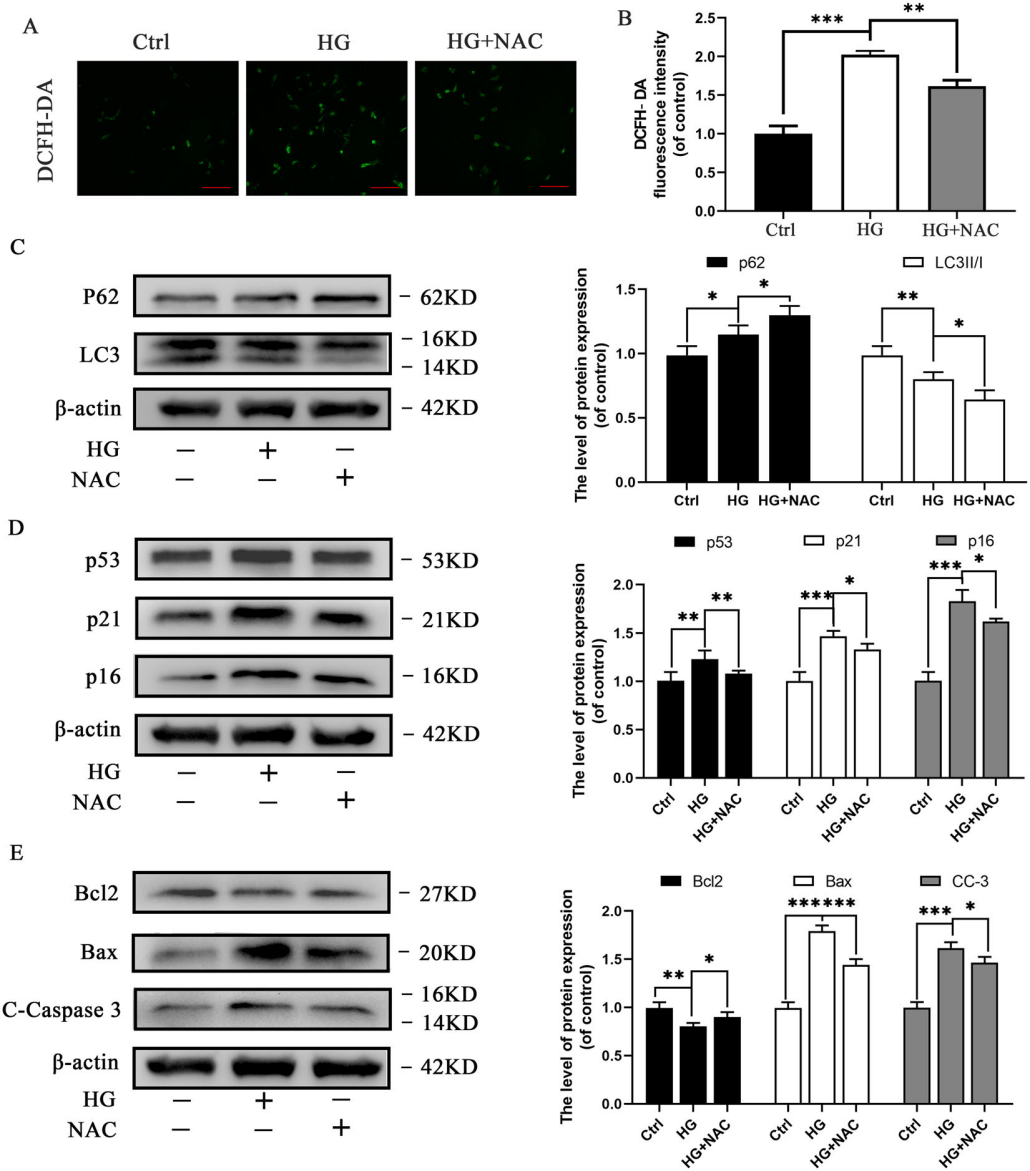
Elimination of oxidative stress suppresses autophagy and inhibits apoptosis and senescence in chondrocytes. **A** Representative micrographs of DCFH-DA staining representing variations in ROS levels upon treatment with NAC. Scale bar= 50 μM. **B** Decrease in DCFH-DA fluorescence intensity in HG-stimulated chondrocytes upon treatment with NAC. **C** Western blot results and quantification analyses of p62 and LC3 in various treatment groups. **D** Western blot results and quantification analyses of p53, p21, and p16 in various treatment groups. **E** Western blot results and quantification analyses of Bcl2, Bax, and cleaved caspase-3 in various treatment groups. Data are presented as means ± SD (*n* = 5). ^*^P < 0.05, ^**^P < 0.01^,^ and ^***^P < 0.001.

NAC is an antioxidant that has been used to prevent the production of ROS in chondrocytes^35^. We initially detected the cytotoxicity of NAC in chondrocytes at various glucose concentrations using the Cell Counting Kit-8 (CCK-8) assay after 24 h of treatment with NAC (Supplementary 1A). The CCK-8 assay results demonstrated that treatment of chondrocytes with NAC for 24 h exerted a negative effect on the viability of chondrocytes over the concentration of 1 mM; therefore, 1 mM NAC was used in subsequent experiments. We evaluated ROS levels in HG-treated chondrocytes in combination with or without NAC to elucidate the role of NAC in HG induced chondrocytes. The results revealed that DCFH-DA positive cells with green fluorescence and DCFH-DA fluorescence intensity increased substantially under HG induced oxidative stress, which was reversed by the administration of NAC (Fig. 3A, B). The results demonstrated that NAC can significantly reduce oxidative stress in HG induced rat chondrocytes.

ROS production as an indicator of oxidative stress is widely recognized as a key mediator of autophagy^33,36^. The increase in ROS level can promote autophagy activation. We evaluated the expression levels of p62 and LC3II/I to explore the effect of oxidative stress elimination on autophagy in HG induced rat chondrocytes (Fig. 3C). Western blot results revealed that autophagy was suppressed in HG induced chondrocytes, and further suppressed upon treatment with NAC, suggesting that elimination of oxidative stress (NAC) suppresses autophagy in HG induced chondrocytes. In addition, the effect of NAC on apoptosis and senescence was evaluated. The levels of senescence-related markers (p53, p21, and p16) and classical pro-apoptotic markers (C-caspase-3 and Bax) were downregulated in NAC-treated groups. The anti-apoptotic index (Bcl-2) increased (Fig. 3D, E). The results suggest that elimination of oxidative stress (NAC) suppresses autophagy, and can inhibit apoptosis and senescence in HG induced rat chondrocytes.

### Activation of AMPK promotes autophagy and suppresses apoptosis and senescence in HG-treated chondrocytes

**Fig. 4:**
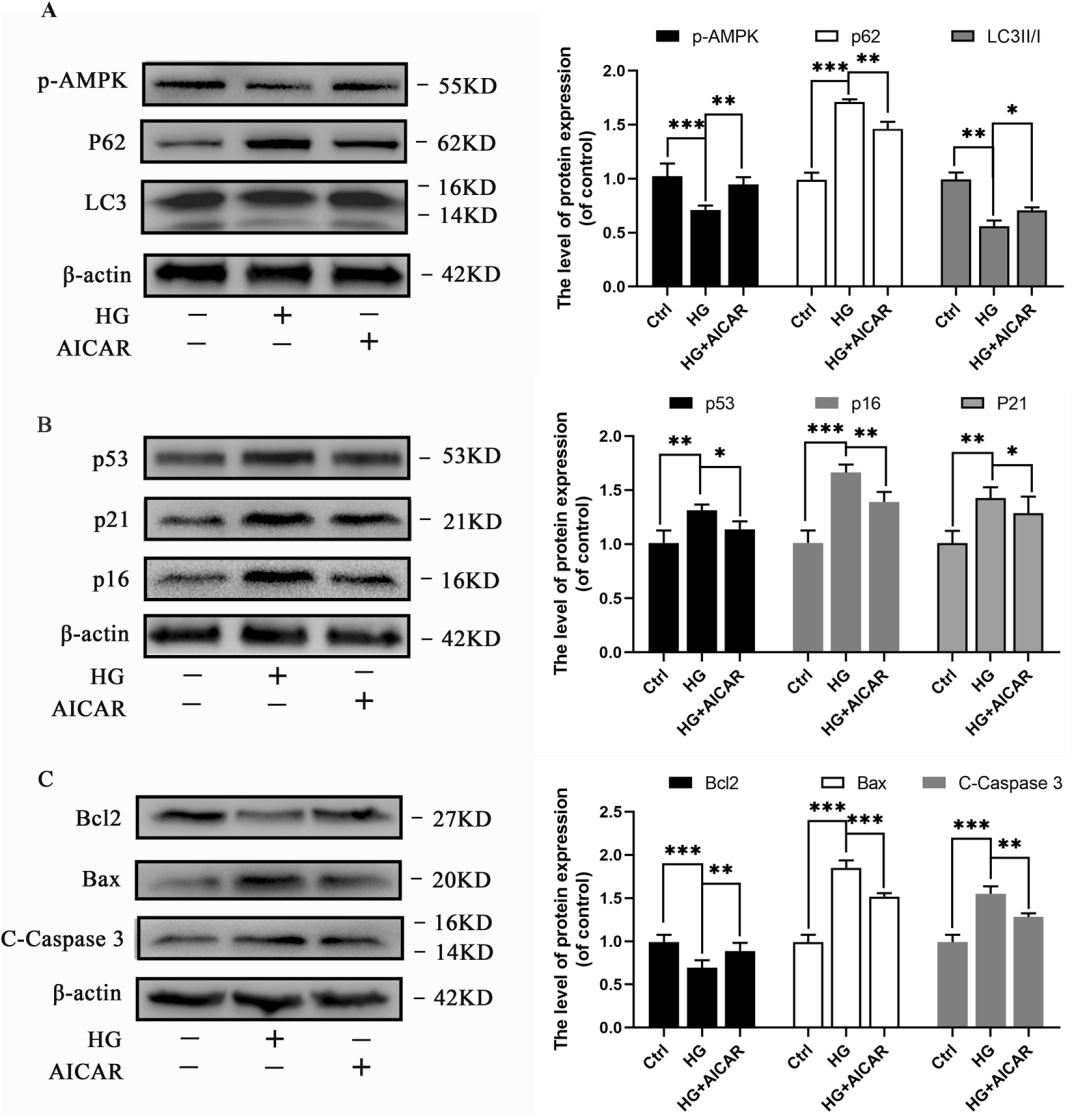
Activation of AMPK promotes autophagy and suppresses apoptosis and senescence in chondrocytes. **A** Western blot results and quantification analyses of p-AMPK, p62, and LC3 in various treatment groups. **B** Western blot results and quantification analyses of p53, p21, and p16 in various treatment groups. **C** Western blot results and quantification analyses of Bcl2, Bax, and cleaved caspase-3 in various treatment groups. Data are presented as means ± SD (*n* = 5). ^*^P < 0.05, ^**^P < 0.01^,^ and ^***^P < 0.001.

AICAR has the capacity to activate AMPK in chondrocytes^37^; therefore, we used AICAR to assess the effect of AMPK activation on autophagy, as well as its cytoprotective effects against apoptosis and senescence in chondrocytes. We initially evaluated the cytotoxicity of AICAR at various concentrations in chondrocytes using CCK-8 assay after 24 h of treatment with AICAR (Supplementary 1B). The CCK-8 assay results demonstrated that a concentration of 1 mM AICAR was the optimum concentration for chondrocytes. HG-stimulated cells were either treated with or without AICAR to explore the capacity of AICAR to activate AMPK in rat chondrocytes. Western blot analyses results revealed that HG decreased the phosphorylation level of AMPK, whereas AICAR increased the phosphorylation level of AMPK, suggesting that AICAR has the capacity to activate AMPK in HG induced chondrocytes (Fig. 4A). A decreased expression of p62 and increased expression of LC3II/I were observed in AICAR-treated groups, which suggested that activation of AMPK promoted autophagy in HG induced chondrocytes (Fig. 4A). Furthermore, we established that the levels of senescence-related and pro-apoptotic markers were increased, whereas Bcl-2 level decreased in AICAR-treated groups when compared with the HG treated group (Fig. 4B, C). The results suggested that activation of AMPK (AICAR treatment) promotes autophagy, and suppresses apoptosis and senescence in HG induced chondrocytes.

### Combined treatment of NAC and AICAR is superior to treatment with either NAC or AICAR

#### In vitro experiments

**Fig. 5:**
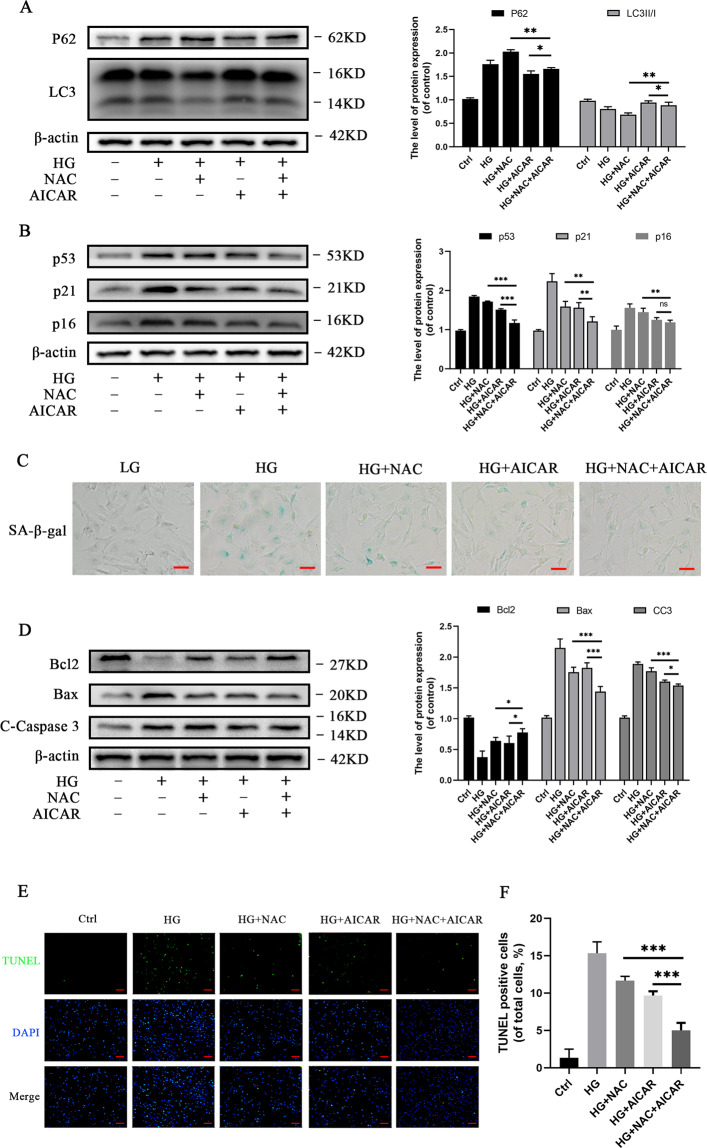
Dual modulation effects of oxidative stress and AMPK (NAC + AICAR) suppresses apoptosis and senescence (2 + 2DG) in chondrocytes. **A** Western blot results and quantification analyses of p62 and LC3 in various treatment groups. **B** Western blot results and quantification analyses of p53, p21, and p16 in various treatment groups. **C** Representative micrographs of SA‐β‐gal staining of chondrocytes in various treatment groups. Scale bar = 50 μM. **D** Western blot results and quantification analyses of Bcl2, Bax, and cleaved caspase-3 in various treatment groups. **E** Representative micrographs of TUNEL staining of chondrocytes to evaluate effects on apoptosis in various treatment groups. Scale bar= 100 μM. **F** TUNEL-positive cell intensity in various treatment groups. Data are presented as means ± SD (*n* = 5). ^*^P < 0.05, ^**^P < 0.01, and ^***^P < 0.001.

Cells were treated with a combination of NAC and AICAR to evaluate the capacity of dual modulation effects of oxidative stress and AMPK on chondrocytes. Western blot analyses results revealed that p62 expression levels decreased and LC3II/I expression levels increased in the HG-treated group when compared to the NAC + AICAR-treated group, which demonstrated an overall activation of autophagy (Fig. 5A). In addition, autophagy in the NAC + AICAR-treated group decreased when compared with the NAC-treated group but increased autophagy when compared with the AICAR-treated group. The levels of senescence-related and apoptosis-related markers decreased, while Bcl-2 level increased in the NAC + AICAR-treated group when compared with the NAC group (Fig. 5B–E). Similar results were observed in the AICAR group except for p16. Strikingly, anti-senescence and anti-apoptotic effects were greater in the AICAR group than in the NAC group. Generally, the results suggested that combined treatment of NAC and AICAR promoted autophagy, and suppressed apoptosis and senescence in HG induced rat chondrocytes; combined treatment of NAC and AICAR was superior to treatment with either NAC or AICAR in vitro.

#### In vivo experiments

**Fig. 6:**
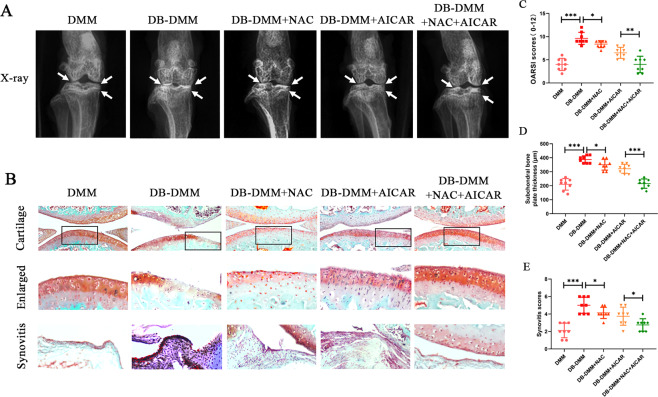
NAC and AICAR ameliorate DB-accelerated OA development in a DB-DMM mouse model in vivo. **A** Digital X-ray image of mice knee joints in the various experimental groups. Joint space, calcification of the cartilage surface, and osteophyte formation are represented by white arrows. **B** Representative Safranin-O staining of cartilage and synovitis in various experimental groups at 4 weeks post surgery. **C** OARSI scores of cartilage. **D**. Subchondral bone plate thicknesses. **E** Synovitis scores. Data are presented as means ± SD (*n* = 8). ^*^P < 0.05, ^**^P < 0.01 and, ^***^P < 0.001.

To investigate the protective effects of NAC and AICAR on DB-accelerated OA in vivo, the DB–OA model was established. The X-ray revealed that the DB-DMM group exhibited formation of severe osteophytes, narrowing of joint spaces, and an increase in cartilage surface density when compared with the DMM group. However, the pathological changes were mild in the treatment groups and the combined treatment was superior to treatment with either NAC or AICAR (Fig. 6A). Histological analyses were performed by staining mice cartilage with safranin O (Fig. 6B). The OARSI scores of the five groups were as follows: DMM = 4.000 ± 1.309, DB-DMM = 9.625 ± 1.302, DB-DMM + NAC = 8.375 ± 0.744, DB-DMM + AICAR = 6.500 ± 1.195, and DB-DMM + NAC + AICAR = 4.000 ± 1.773 (Fig. 6C). The subchondral bone thicknesses in the five groups were as follows: DMM = 210.0 ± 41.4, DB-DMM = 387.5 ± 26.1, DB-DMM + NAC = 350.0 ± 38.5, DMM + AICAR = 321.3 ± 32.3, and DMM + NAC + AICAR = 215.0 ± 3.4 (Fig. 6D). Synovial thickening and hypercellularity in the five groups were as follows: DMM = 2.125 ± 0.835, DB-DMM = 5.000 ± 0.926, DMM + NAC = 4.125 ± 0.641, DMM + AICAR = 3.750 ± 1.035, and DMM + NAC + AICAR = 2.750 ± 0.707 (Fig. 6E). The results suggested that NAC and AICAR ameliorated DB–OA progression in vivo and that the combined treatment of NAC and AICAR was superior to treatment with either NAC or AICAR in vivo.

## Discussion

OA is a chronic and widespread disease, which is a principal cause of disability among the elderly, in turn, resulting in high social costs. As the population ages and the population with DB increases, OA has increasingly become prevalent over the last few decades^19,38^. Epidemiological studies conducted recently and experimental evidence have demonstrated that DB can induce the occurrence of OA and exacerbate the burden associated with OA^1,39,40^. Diabetic patients with OA are prescribed non-steroidal anti-inflammatory drugs or muscle relaxants to relieve symptoms; however, few drugs and treatment strategies are available for DB–OA progression. Presently, a few studies on the underlying mechanisms of DB or HG that influence OA progression have been performed. The present study revealed that HG can suppress autophagy through the AMPK pathway and induce autophagy via oxidative stress in chondrocytes in vitro. In addition, NAC and AICAR can ameliorate DB–OA progression in vivo, and the therapeutic effect of combined treatment was superior to individual drug treatments.

Alteration of autophagy-related proteins leads to the destruction of the articular cartilage that ultimately causes entire joint damage and the development of OA^18^. HG can considerably influence the level of autophagy; however, the results were inconsistent. A study by Ribeiro et al. revealed that HG inhibited autophagy to accelerate cartilage degradation, and pharmacological autophagy activation inversely attenuated the harmful effects on chondrocytes^22^. Adversely, other studies reported that autophagy as a response mechanism was upregulated to offer protection against HG-induced apoptosis and senescence, which means completely reverse result^21,26,41^. In the present study, the results of in vitro experiments revealed that autophagy levels changed dynamically with increase in glucose concentration. Therefore, we hypothesized that HG influenced autophagy via two distinct mechanisms at various glucose concentrations. The first hypothesis was that HG decreased autophagy to negatively influence chondrocytes via a certain pathway, and the second hypothesis was that HG increased autophagy as a response mechanism to protect from cellular damage via a different pathway. Therefore, the present study focused on investigating the two mechanisms involved in autophagy.

In the late 1990s, the “radical free” concept was posited^42^. Living cells can be influenced by the harmful effects of exogenously or endogenously produced high concentrations of reactive oxidizing molecules^29,30^. Among the molecules, ROS has been identified as a key oxidizing molecule with biological influence^36^. HG, which is a risk factor for DB, can rapidly increase energetic stress in cells, cause mitochondrial overburden and electron leakage, and eventually increase the production of oxidized molecules.^36^HG can generate oxidized molecules to induce oxidative stress and HG-induced oxidative stress results in cell damage^29,43–45^. Our results were consistent with the findings of previous studies. The level of HG-produced ROS in chondrocytes activated apoptosis and senescence in a concentration-depend manner, which was significantly reversed by the antioxidant, NAC.

In addition, oxidative stress is a key factor influencing autophagy^33,46–48^, and ROS has been considered an early inducer of autophagy^49^. Superoxide anion (O_2_^−^) and H_2_O_2_ are the principal forms of ROS produced by various stimulants including HG and are crucial in the activation of autophagy because treatment with antioxidants partially or completely reverses the effect^50^. According to a previous study, elevated ROS level was observed in axons after hypoxic-ischemic injury, and activation of autophagy in the striatum and cortex was observed in adult and neonatal mice^51^. Another study revealed that ROS following cerebral hypoxia-ischemia induced autophagy to eliminate injured mitochondria and prevent necrosis^52,53^, which was consistent with the findings of the present study. The present study demonstrated that HG stimulated oxidative stress, which in turn, induced the production of ROS, although the decrease in autophagy was further enhanced by NAC, implying that HG partially increased autophagy in chondrocytes via oxidative stress. Therefore, autophagy, which functioned as a feedback regulation mechanism, was considered to be a protective mechanism associated with HG stimulation. Although the specific mechanisms were not elucidated in this study, activation of autophagy by ROS could be attributed to the following reasons. First, ROS is transduced by a ROS-producing system located in or adjacent to the plasma membrane, such as the NADPH oxidase (NOX) complexes. ROS produced by NOX2 are essential for the recruitment of microtubule-associated protein LC3 on phagosomes^54^. Second, mitochondria are the primary sites of ROS generation and are the organelles with the capacity to activate and regulate autophagy; thus, mitophagy could play a pivotal role in cellular autophagy. The impairment of mitochondrial function caused by HG in cells results in the production of large quantities of ROS, which suggests that mitophagy is a key factor influencing response to oxidative stress. The mitochondria-selective process of mitophagy can be modulated by certain proteins, including BNIP3 (BCL2/adenovirus E1B 19 kDa interacting protein 3), PARK2, PINK1 (PTEN-induced putative kinase 1), and mitochondrial fusion and fission processes^55–57^. Finally, ROS, especially H_2_O_2_, can bind to the cysteine 81 site of ATG4 to directly activate ATG4 and lipidation of LC3^33,58^, which is a vital process for the formation of autophagosomes. After autophagosomes mature and fuse with lysosomes, ATG4 delipidates LC3. However, H_2_O_2_-mediated oxidation of ATG4 can inactivate delipidization of LC3 during the process. A detailed study by Rodriguez-Muela and colleagues demonstrated that *Atg4B*^*−/−*^ mice exhibited a marked reduction of LC3 and an increased SQSTM1 level^59^.

AMPK is a genuine sensor of the energy status of cells, which can be inhibited in HG environments^34,60,61^. At the molecular level, the AMPK signaling pathway is extensively considered to be a key factor influencing the stimulation of autophagy. In the present study, the AMPK signaling pathway and autophagy were significantly inhibited, and apoptosis-related and senescence-related markers were significantly upregulated when chondrocytes were treated with HG concentrations; the phenomena were reversed by AICAR. The results suggest that HG causes apoptosis and senescence in chondrocytes by inhibiting autophagy through the AMPK signaling pathway.

Additionally, there could have been a crosstalk between oxidative stress and the AMPK pathway when HG stimulated chondrocytes at a certain glucose concentration. Particularly, the regulation of ROS as a product of oxidative stress in the AMPK pathway in various types of cells has been contradictory. A few researchers have argued that oxidative stress can damage the mitochondrial structure, which results in energy imbalance. AMPK is a crucial sensor of cellular energy that can be activated by ROS^53^ and mTOR is subsequently inhibited to activate autophagy. However, a study by Jiang et al. demonstrated that ROS induced by HG facilitated the recruitment of MG53 and subsequent degradation of p-AMPKα at S485/491^62^. Moreover, HG deactivated AMPK, which was associated with ROS-dependent suppression of AMPKα phosphorylation at T172 rather than ATP level^62^. According to the results of the present study, the AMPK pathway was inhibited by HG, which implied that ROS did not activate AMPK to increase autophagy. Furthermore, ROS was demonstrated to increase autophagy, which suggested that ROS-dependent suppression of AMPK was insignificant or did not occur. Therefore, the crosstalk may not be vital in the regulation of autophagy in HG-stimulated chondrocytes.

**Fig. 7:**
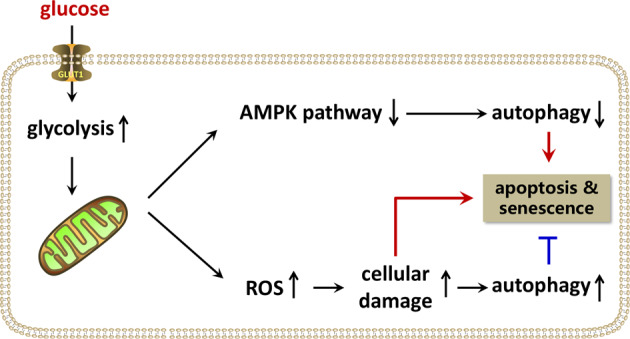
A schematic diagram of the dual effect of high glucose on autophagy in chondrocytes. High glucose suppresses autophagy through the AMPK pathway while it induces autophagy via oxidative stress in chondrocytes.

Overall, the present study revealed that HG-induced autophagy is the synthetic results via two different mechanisms; the first mechanism is autophagy activation by oxidative stress and the second mechanism is autophagy inhibition via the AMPK signaling pathway (Fig. 7). AMPK signaling pathway plays a major role in decreasing autophagy in chondrocytes relative to oxidative stress. The effect of oxidative stress associated with autophagy activation is significantly enhanced with an increase in glucose concentration; therefore, the overall autophagy was inversely upregulated.

The present study provides a novel strategy for treating DB-accelerated OA. Although several researchers have focused on the pharmacological activation of autophagy via the AMPK signaling pathway to attenuate adverse effects of HG, pharmacological remission of oxidative stress should not be overlooked. In vivo and in vitro experiments revealed that NAC and AICAR effectively alleviated senescence and apoptosis in HG-induced chondrocytes. Moreover, combined treatment of NAC and AICAR was superior to treatment with either NAC or AICAR. We observed that the role of AICAR seemed superior to that of NAC and that the p16 level in the NAC + AICAR group was significantly lower than that in the NAC group, although not significantly different when compared with the AICAR group. The observations could be because AICAR not only increased autophagy to alleviate senescence and apoptosis but also ameliorated oxidative stress based on increased autophagy to enhance its roles. Furthermore, the combined application of other antioxidants and AMPK activation agents to replace these drugs can be an alternative.

The present study had a few limitations. First, we did not investigate the specific mechanisms associated with the two pathways in detail; therefore, the specific molecular changes were obscure except for p-AMPK. In addition, the specific mechanisms elucidating the role of ROS in autophagy remain unknown. Second, the long-term therapeutic effects and side effects of the combined treatment in DB-accelerated OA are indeterminate and require further study.

## Conclusion

The present study has demonstrated that high glucose suppresses autophagy through the AMPK signaling pathway and induces autophagy via oxidative stress in chondrocytes. The combined treatment of NAC and AICAR is a novel and potential strategy for treating DB-accelerated OA.

## Materials and methods

### Reagents and antibodies

Primary antibodies including p62 (ab109012), LC3 (ab62721), B-cell lymphoma 2 (Bcl-2, ab196495), p16 (ab51243), p21(ab109199) and β-actin (ab6276) were acquired from Abcam (Cambridge, MA, USA). Primary antibodies against p-AMPK (50081), AMPK (5831), Bax (2772), and p53 (2524) were purchased from Cell Signaling Technologies (CST; Danvers, MA, USA). Primary antibodies against cleaved caspase-3 (C-caspase-3, WL01992) were purchased from WanleiBio (Shen Yang, China). Goat anti-rabbit IgG (7074) and goat anti-mouse IgG (7076) secondary antibodies were purchased from CST (Danvers, MA, USA). Cell culture reagents were purchased from Gibco (Grand Island, NY, USA). D-(+)-Glucose was purchased from Sigma-Aldrich (St. Louis, MO, USA). AICAR and Acetylcysteine also called N-acetyl cysteine (NAC) were acquired from MedChemExpress (MCE; Manmouth Junction, NJ, USA).

### Isolation and culture of rat chondrocytes

Sprague–Dawley rat articular cartilage was collected from the underlying bone and connective tissues of 5-day-old rats. The cartilage tissues were cut into 1 × 1 × 1 mm^3^ pieces and washed three times with phosphate-buffered saline (PBS). The tissues were subsequently digested in 0.2% type II collagenase (Sigma-Aldrich, St. Louis, MO, USA) for 4 h at 37 °C. After washing with PBS, the digested tissues were transferred into a mixed medium containing 1 g/L D-Glucose, which included Dulbecco’s modified Eagle’s medium (DMEM)/F12 (Gibco, Invitrogen, Grand Island, NY, USA) with 15% fetal bovine serum (FBS; Gibco, Invitrogen, Grand Island, NY, USA) and antibiotics (1% penicillin-streptomycin), and incubated in 5% CO_2_ at 37 °C. When confluent, the cells were passaged after treatment with 0.25% trypsin-EDTA (Gibco, Invitrogen, Grand Island, NY, USA) and reseeded into 10-cm culture plates at the appropriate density under a humidified atmosphere. We only used passages three or four in our experiments.

### Experimental design

#### In vitro experiments

Normal rat chondrocytes were cultured in glucose concentration of 5.6 mM, which was similar to the normal blood sugar level. Chondrocytes were incubated at different glucose concentrations (12.5, 25, and 50 mM) to simulate HG stimulation in vitro. In addition, chondrocytes treated with a glucose concentration of 50 mM were selected as the HG group in subsequent analyses. The group treated with 50 mM HG was either treated with or without AICAR (1 mM), with or without NAC (1 mM). Cells were harvested after 24 h of incubation.

#### In vivo experiments

Male C57BL/6 mice (8 weeks old) were randomly divided into five groups with 8 mice in each group, and the selection of sample size for animal experiments is carried out as per the preliminary experiments as well as similar well-designed experiments^22^. The mice were operated through surgical destabilization of the medial meniscus (DMM): with AICAR), and group E (DMM treated with NAC and AICAR). After fasting for 10 h, the mice were injected intraperitoneally with 0.1 mol/L streptozotocin (STZ) at a dose of 100 mg/kg/day until blood glucose concentration was >16.7 mmol/L, which should be stable for at least 5 days. Afterward, the mice were fed with high-fat and high-sugar diets (HFSD) for 4 weeks to establish DB models. The DB mice subjected to DMM were considered as the DB-accelerated OA models (DB-OA models)^22^. Mice in the remaining groups except for the control group were fed with HFSD. Mice in the treatment groups were injected intraperitoneally with AICAR at a dose of 200 mg/kg/day or received an intro-articular injection of NAC at a dose of 5 mg/kg/3 days based on previous studies^63–65^. All mice were euthanized 4 weeks after surgery and the cartilage tissues were used for iconographic and histological analyses.

### Western blot assay

Chondrocytes were lysed in ice-cold radioimmunoprecipitation assay buffer with 1 mM phenylmethanesulfonyl fluoride and centrifuged at 4 °C and 12,000 rpm. The protein concentrations were quantified using the BCA Protein Assay Kit (Beyotime Biotechnology, Shanghai, China). Protein samples were separated using sodium dodecylsulfate-polyacrylamide gel electrophoresis and subsequently transferred to polyvinylidene difluoride membranes (Bio-Rad, Hercules, CA, USA). The membranes were blocked with 5% nonfat milk for 1.5 h and probed with primary antibodies against p62 (1:1000), LC3 (1:1000), β-actin (1:2000), p-AMPK (1:1000), C-caspase-3 (1:500), Bax (1:1000), Bcl-2 (1:1000), p53 (1:1000), p21 (1:1000), and p16 (1:1000) overnight at 4 °C before incubating with the respective secondary antibodies for 2 h at room temperature. Finally, the intensities of protein bands were quantified using Image Lab software (version 3.0; Bio-Rad, Hercules, CA, USA).

### Senescence–associated β-galactosidase (SA-β-gal) staining

Cells were fixed on plates with 0.2% glutaraldehyde for 15 min after washing them three times with PBS. Subsequently, cells were stained using an X-gal staining solution at a pH of 6.0 and incubated overnight. The images were captured by Olympus IX71 microscope (Olympus Inc., Tokyo, Japan). Finally, the percentages of SA-β-gal-positive cells were counted for subsequent statistical analysis.

### TUNEL assay

DNA fragmentations resulting from apoptosis were measured using the terminal deoxynucleotidyl transferase (TdT) dUTP nick-end labeling (TUNEL) assay. After fixing with 4% paraformaldehyde for 1 h, cells were incubated with 3% hydrogen peroxide (H_2_O_2_) and 0.1% Triton X-100 for 10 min. The cells were stained using the In Situ Cell Death Detection Kit (F. Hoffmann-LaRoche Ltd., Basel, Switzerland) and 4, 6-diamidino-2-phenylindole (DAPI). Finally, slides were imaged using a fluorescence microscope (Olympus Inc., Tokyo, Japan).

### Reactive oxygen species assay

The reactive oxygen species (ROS) level in cells were measured using 2′,7′-dichlorodihydro-fluorescein diacetate (DCFH-DA) Staining Assay Kit (Beyotime Biotechnology, Shanghai, China). Chondrocytes in plates were incubated with DCFH-DA mixed basic culture medium (1:1000) for 30 min based on the previous experimental procedures^66^. Finally, the random fields were captured by fluorescence microscopy (Olympus Inc., Tokyo, Japan). The results of relative fluorescence unit (RFU) values were calculated by subtracting the blank well values from the RFU values of the assay wells, which were detected at 490/520 nm by a multifunction fluorescence microplate reader.

### X-ray imaging

Mice in five groups were examined with X-ray 4 weeks after surgery to assess the joint spaces, osteophyte formation, and calcification changes using a digital X-ray machine (Kubtec Model XPERT.8; KUB Technologies Inc.). Suitable images were obtained in the following settings: 50 kV and 160 μA.

### Histopathological analysis

The joint tissues of mice were fixed with 4% paraformaldehyde for 2 days and decalcified in neutral 10% EDTA solution for 2 weeks. Subsequently, the samples were cut into 5-µm slides. The slides were deparaffinized in xylene and rehydrated in ethanol washes. Slides with joint tissues from five groups were subjected to safranin O-fast green (S–O) staining. The cellularity and morphology of the cartilages were blindly evaluated by three experienced histology researchers using the Osteoarthritis Research Society International (OARSI) scoring system as described previously^67^. The synovitis and subchondral cortical bone thickness were graded as described previously^5,68,69^. The AxioVision software (Zeiss, Oberkochen, Germany) was used to measure the thickness of the medial subchondral bone plate.

### Statistical analysis

Statistical analyses were performed using SPSS statistical software version 22.0 (SPSS, Inc., Chicago, IL, USA). All experiments were repeated at least five times. The results were presented as means ± standard deviation (SD). For parametric data, the differences among four groups were analyzed by one-way analysis of variance (ANOVA), and the differences between the two groups were analyzed by *t-*test. The nonparametric data were analyzed using the Kruskal–Wallis *H* test. *P* value < 0.05 was considered statistically significant.

## Supplementary information

Supplement Material

## References

[1] Litwic A, Edwards MH, Dennison EM, Cooper C (2013). Epidemiology and burden of osteoarthritis. Br. Med. Bull..

[2] Blagojevic M, Jinks C, Jeffery A, Jordan KP (2010). Risk factors for onset of osteoarthritis of the knee in older adults: a systematic review and meta-analysis. Osteoarthr. Cartil..

[3] Sun MM, Beier F, Pest MA (2017). Recent developments in emerging therapeutic targets of osteoarthritis. Curr. Opin. Rheumatol..

[4] Gu YT (2017). Research progress on osteoarthritis treatment mechanisms. Biomed. Pharmacother..

[5] Lewis JS (2011). Acute joint pathology and synovial inflammation is associated with increased intra-articular fracture severity in the mouse knee. Osteoarthr. Cartil..

[6] Arellano Perez Vertti RD (2019). Cartilage oligomeric matrix protein levels in Type 2 diabetes associated with primary knee osteoarthritis patients. Genet. Test. Mol. Biomark..

[7] King KB, Rosenthal AK (2015). The adverse effects of diabetes on osteoarthritis: update on clinical evidence and molecular mechanisms. Osteoarthr. Cartil..

[8] Cohen MP, Surma ML (1984). Effect of diabetes on in vivo metabolism of [35S]-labeled glomerular basement membrane. Diabetes.

[9] Umpierrez GE, Zlatev T, Spanheimer RG (1989). Correction of altered collagen metabolism in diabetic animals with insulin therapy. Matrix.

[10] Silberberg R, Hirshberg GE, Lesker P (1977). Enzyme studies in the articular cartilage of diabetic rats and of rats bearing transplanted pancreatic islets. Diabetes.

[11] Kayal RA (2007). Diminished bone formation during diabetic fracture healing is related to the premature resorption of cartilage associated with increased osteoclast activity. J. Bone Min. Res..

[12] Alblowi J (2013). Chemokine expression is upregulated in chondrocytes in diabetic fracture healing. Bone.

[13] Pessler F (2008). The synovitis of “non-inflammatory” orthopaedic arthropathies: a quantitative histological and immunohistochemical analysis. Ann. Rheum. Dis..

[14] Guariguata L (2014). Global estimates of diabetes prevalence for 2013 and projections for 2035. Diabetes Res. Clin. Pr..

[15] Vos T (2012). Years lived with disability (YLDs) for 1160 sequelae of 289 diseases and injuries 1990–2010: a systematic analysis for the Global Burden of Disease Study 2010. Lancet.

[16] Giampieri F (2019). Autophagy in human health and disease: novel therapeutic opportunities. Antioxid. Redox Signal..

[17] Abounit K, Scarabelli TM, McCauley RB (2012). Autophagy in mammalian cells. World J. Biol. Chem..

[18] Caramés B, Taniguchi N, Otsuki S, Blanco FJ, Lotz M (2010). Autophagy is a protective mechanism in normal cartilage, and its aging-related loss is linked with cell death and osteoarthritis. Arthritis Rheum..

[19] Goldring MB, Goldring SR (2007). Osteoarthritis. J. Cell. Physiol..

[20] Levine B, Kroemer G (2008). Autophagy in the pathogenesis of disease. Cell.

[21] Jiang L (2013). Apoptosis, senescence, and autophagy in rat nucleus pulposus cells: implications for diabetic intervertebral disc degeneration. J. Orthop. Res..

[22] Ribeiro M (2016). Diabetes-accelerated experimental osteoarthritis is prevented by autophagy activation. Osteoarthr. Cartil..

[23] Ribeiro M, López de Figueroa P, Blanco FJ, Mendes AF, Caramés B (2016). Insulin decreases autophagy and leads to cartilage degradation. Osteoarthr. Cartil..

[24] Zhou DM (2020). Metformin inhibits high glucose-induced smooth muscle cell proliferation and migration. Aging.

[25] Chae CW (2020). High glucose-mediated PICALM and mTORC1 modulate processing of amyloid precursor protein via endosomal abnormalities. Br. J. Pharm..

[26] Zhao LG (2020). Mibefradil alleviates high-glucose-induced cardiac hypertrophy by inhibiting PI3K/Akt/mTOR-mediated autophagy. J. Cardiovasc. Pharm..

[27] Guo X (2020). 1,25-Dihydroxyvitamin D attenuates diabetic cardiac autophagy and damage by vitamin D receptor-mediated suppression of FoxO1 translocation. J. Nutr. Biochem..

[28] Han D (2020). SIRT3 deficiency is resistant to autophagy-dependent ferroptosis by inhibiting the AMPK/mTOR pathway and promoting GPX4 levels. J. Cell. Physiol..

[29] Luc, K., Schramm-Luc A., Guzik T. J. & Mikolajczyk, T. P. Oxidative stress and inflammatory markers in prediabetes and diabetes. *J. Physiol. Pharmacol.* **70**, 2019. 10.26402/jpp.2019.6.01 32084643

[30] Singh, H. et al. Protective role of Phyllanthusfraternus in alloxan-induced diabetes in rats. *J. Ayurveda Integr. Med.* **11**, 391–398 (2020). 10.1016/j.jaim.2019.09.008 PMC7772496 32088092

[31] Ronnett GV, Ramamurthy S, Kleman AM, Landree LE, Aja S (2009). AMPK in the brain: its roles in energy balance and neuroprotection. J. Neurochem..

[32] Garcia D, Shaw RJ (2017). AMPK: mechanisms of cellular energy sensing and restoration of metabolic balance. Mol. Cell.

[33] Lin WJ, Kuang HY (2014). Oxidative stress induces autophagy in response to multiple noxious stimuli in retinal ganglion cells. Autophagy.

[34] Cardaci S, Filomeni G, Ciriolo MR (2012). Redox implications of AMPK-mediated signal transduction beyond energetic clues. J. Cell Sci..

[35] Yu SM, Kim SJ (2015). The thymoquinone-induced production of reactive oxygen species promotes dedifferentiation through the ERK pathway and inflammation through the p38 and PI3K pathways in rabbit articular chondrocytes. Int. J. Mol. Med..

[36] Filomeni G, De Zio D, Cecconi F (2015). Oxidative stress and autophagy: the clash between damage and metabolic needs. Cell Death Differ..

[37] Husa M, Petursson F, Lotz M, Terkeltaub R, Liu-Bryan R (2013). C/EBP homologous protein drives pro-catabolic responses in chondrocytes. Arthritis Res. Ther..

[38] Caterson B, Baker JR, Christner JE, Pollok BA, Rostand KS (1980). Diabetes and osteoarthritis. Ala. J. Med. Sci..

[39] Berenbaum F (2012). Diabetes-induced osteoarthritis: from a new paradigm to a new phenotype. Postgrad. Med. J..

[40] Schwarz S, Mrosewski I, Silawal S, Schulze-Tanzil G (2018). The interrelation of osteoarthritis and diabetes mellitus: considering the potential role of interleukin-10 and in vitro models for further analysis. Inflamm. Res..

[41] Yang Z (2020). Prokineticin 2 (PK2) rescues cardiomyocytes from high glucose/high palmitic acid-induced damage by regulating the AKT/GSK3β pathway in vitro. Oxid. Med. Cell. Longev..

[42] Jones DP (2008). Radical-free biology of oxidative stress. Am. J. Physiol. Cell Physiol..

[43] Pal S, Rao GN, Pal A (2020). High glucose-induced ROS accumulation is a critical regulator of ERK1/2-Akt-tuberin-mTOR signalling in RGC-5 cells. Life Sci..

[44] Zhang W., Sui Y. CircBPTF knockdown ameliorates high glucose-induced inflammatory injuries and oxidative stress by targeting the miR-384/LIN28B axis in human umbilical vein endothelial cells. *Mol. Cell. Biochem.* **471**, 101–111 (2020). 10.1007/s11010-020-03770-2 32524321

[45] Li R (2017). NGF attenuates high glucose-induced ER stress, preventing Schwann cell apoptosis by activating the PI3K/Akt/GSK3β and ERK1/2 pathways. Neurochem. Res..

[46] Hariharan N, Zhai P, Sadoshima J (2011). Oxidative stress stimulates autophagic flux during ischemia/reperfusion. Antioxid. Redox Signal..

[47] Cimini S, Rizzardini M, Biella G, Cantoni L (2014). Hypoxia causes autophagic stress and derangement of metabolic adaptation in a cell model of amyotrophic lateral sclerosis. J. Neurochem..

[48] Essick EE, Sam F (2010). Oxidative stress and autophagy in cardiac disease, neurological disorders, aging and cancer. Oxid. Med. Cell. Longev..

[49] Filomeni G, Desideri E, Cardaci S, Rotilio G, Ciriolo MR (2010). Under the ROS…thiol network is the principal suspect for autophagy commitment. Autophagy.

[50] Levonen AL, Hill BG, Kansanen E, Zhang J, Darley-Usmar VM (2014). Redox regulation of antioxidants, autophagy, and the response to stress: implications for electrophile therapeutics. Free Radic. Biol. Med..

[51] Zhu C (2006). Different apoptotic mechanisms are activated in male and female brains after neonatal hypoxia-ischaemia. J. Neurochem..

[52] Almasieh M (2012). The molecular basis of retinal ganglion cell death in glaucoma. Prog. Retinal Eye Res..

[53] Adhami F, Schloemer A, Kuan CY (2007). The roles of autophagy in cerebral ischemia. Autophagy.

[54] Huang J (2009). Activation of antibacterial autophagy by NADPH oxidases. Proc. Natl. Acad. Sci. USA.

[55] Schweers RL (2007). NIX is required for programmed mitochondrial clearance during reticulocyte maturation. Proc. Natl. Acad. Sci. USA.

[56] Tracy K (2007). BNIP3 is an RB/E2F target gene required for hypoxia-induced autophagy. Mol. Cell. Biol..

[57] Narendra D, Tanaka A, Suen DF, Youle RJ (2008). Parkin is recruited selectively to impaired mitochondria and promotes their autophagy. J. Cell Biol..

[58] Scherz-Shouval R (2007). Reactive oxygen species are essential for autophagy and specifically regulate the activity of Atg4. EMBO J..

[59] Rodríguez-Muela N, Germain F, Mariño G, Fitze PS, Boya P (2012). Autophagy promotes survival of retinal ganglion cells after optic nerve axotomy in mice. Cell Death Differ..

[60] Hardie DG (2011). AMP-activated protein kinase: an energy sensor that regulates all aspects of cell function. Genes Dev..

[61] Oakhill JS (2011). AMPK is a direct adenylate charge-regulated protein kinase. Science.

[62] Jiang, P. et al. Negative regulation of AMPK signaling by high glucose via E3 ubiquitin ligase MG53. *Mol. Cell* **81***,* 629–637 (2021). 10.1016/j.molcel.2020.12.008 33400924

[63] Nakagawa S (2010). N-acetylcysteine prevents nitric oxide-induced chondrocyte apoptosis and cartilage degeneration in an experimental model of osteoarthritis. J. Orthop. Res..

[64] Liu TY (2016). FNDC5 alleviates hepatosteatosis by restoring AMPK/mTOR-mediated autophagy, fatty acid oxidation, and lipogenesis in mice. Diabetes.

[65] Pigna E (2016). Aerobic exercise and pharmacological treatments counteract cachexia by modulating autophagy in colon cancer. Sci. Rep..

[66] Wang, K. et al Genistein protects intervertebral discs from degeneration via Nrf2-mediated antioxidant defense system: An in vitro and in vivo study. *J. Cell. Physiol.*10.1002/jcp.28301 (2019). 10.1002/jcp.28301 30779107

[67] Glasson SS, Chambers MG, Van Den Berg WB, Little CB (2010). The OARSI histopathology initiative - recommendations for histological assessments of osteoarthritis in the mouse. Osteoarthr. Cartil..

[68] Permuy M (2015). Effects of diacerein on cartilage and subchondral bone in early stages of osteoarthritis in a rabbit model. BMC Vet. Res..

[69] Goldring SR (2009). Role of bone in osteoarthritis pathogenesis. Med. Clin. North Am..

