# Supplementary material for: High glucose suppresses autophagy through the AMPK pathway while it induces autophagy via oxidative stress in chondrocytes
Source: Cell Death Dis. 2021 May 18;12(6):506. doi: 10.1038/s41419-021-03791-9 (PMC8131591; doi:10.1038/s41419-021-03791-9)
Supplement: Supplementary file 1 — Supplement Material [file 41419_2021_3791_MOESM1_ESM.pdf]

Figure S1

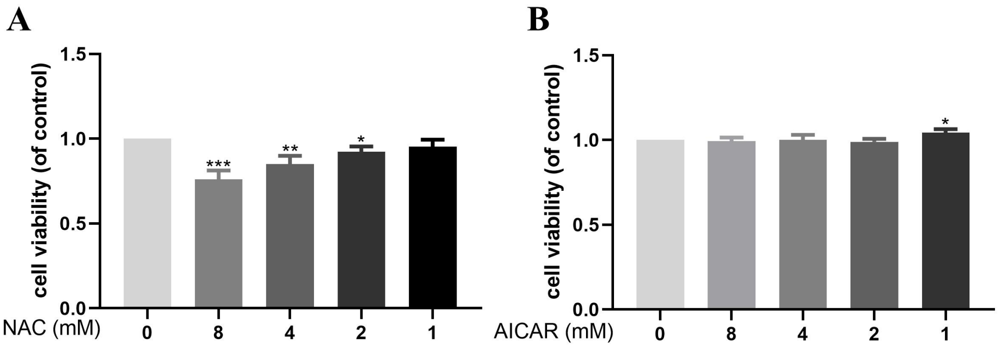

Supplement 1: Effects of NAC and AICAR on cell viability of chondrocytes. (A) Cytotoxic effects of NAC on chondrocytes at various concentrations after 24 hours of treatment administration as determined by the CCK-8 assay. (B) Cytotoxic effects of AICAR on chondrocytes at various concentrations after 24 hours of treatment administration as determined by the CCK-8 assay. Data are presented as means ± SD (n=5). P vs. the control group. $^{*}P < 0.05$, $^{**}P < 0.01$, and $^{***}P < 0.001$
